# Examining the influence of budget execution processes on the efficiency of county health systems in Kenya

**DOI:** 10.1093/heapol/czac098

**Published:** 2022-11-11

**Authors:** Anita Musiega, Benjamin Tsofa, Lizah Nyawira, Rebecca G Njuguna, Joshua Munywoki, Kara Hanson, Andrew Mulwa, Sassy Molyneux, Isabel Maina, Charles Normand, Julie Jemutai, Edwine Barasa

**Affiliations:** Health Economics Research Unit, KEMRI-Wellcome Trust Research Programme, 197 Lenana Place, Lenana Road, P.O Box 43460-00100, Nairobi, Kenya; Institute of Healthcare Management, Strathmore Business School, Strathmore University, Ole Sangale Road, P.O. Box 59857-00200, Madaraka, Nairobi, Kenya; Health Systems and Research Ethics Department, KEMRI-Wellcome Trust Research Programme, P.O Box 230-8010, Kilifi, Kenya; Health Economics Research Unit, KEMRI-Wellcome Trust Research Programme, 197 Lenana Place, Lenana Road, P.O Box 43460-00100, Nairobi, Kenya; Health Economics Research Unit, KEMRI-Wellcome Trust Research Programme, 197 Lenana Place, Lenana Road, P.O Box 43460-00100, Nairobi, Kenya; Health Economics Research Unit, KEMRI-Wellcome Trust Research Programme, 197 Lenana Place, Lenana Road, P.O Box 43460-00100, Nairobi, Kenya; Faculty of Public Health and Policy, London School of Hygiene and Tropical Medicine, Keppel Street, London WC1E 7HT, UK; Directorate of Medical Services, Preventive and Pomotive Health, Ministry of Health, Afya House, Cathedral Road, P.O. Box 30016-00100, Nairobi, Kenya; Health Systems and Research Ethics Department, KEMRI-Wellcome Trust Research Programme, P.O Box 230-8010, Kilifi, Kenya; Health Financing Department, Ministry of Health, Afya House, Cathedral Road, P.O. Box 30016-00100, Nairobi, Kenya; Centre for Health Policy and Management, Trinity College, The University of Dublin, Dublin, Ireland; Health Economics Research Unit, KEMRI-Wellcome Trust Research Programme, 197 Lenana Place, Lenana Road, P.O Box 43460-00100, Nairobi, Kenya; Health Economics Research Unit, KEMRI-Wellcome Trust Research Programme, 197 Lenana Place, Lenana Road, P.O Box 43460-00100, Nairobi, Kenya; Institute of Healthcare Management, Strathmore Business School, Strathmore University, Ole Sangale Road, P.O. Box 59857-00200, Madaraka, Nairobi, Kenya; Centre for Tropical Medicine and Global Health, Nuffield Department of Medicine, University of Oxford, The Peter Medawar Building for Pathogen Research, South Parks Road, Oxon, Oxford OX1 3SY, UK

**Keywords:** Public financial management, budget execution, efficiency

## Abstract

Public financial management (PFM) processes are a driver of health system efficiency. PFM happens within the budget cycle which entails budget formulation, execution and accountability. At the budget execution phase, budgets are implemented by spending as planned to generate a desired output or outcome. Understanding how the budget execution processes influence the use of inputs and the outcomes that result is important for maximizing efficiency. This study sought to explain how the budget execution processes influence the efficiency of health systems, an area that is understudied, using a case study of county health systems in Kenya. We conducted a concurrent mixed methods case study using counties classified as relatively efficient (*n* = 2) and relatively inefficient (*n* = 2). We developed a conceptual framework from a literature review to guide the development of tools and analysis. We collected qualitative data through document reviews and in-depth interviews (*n* = 70) with actors from health and finance sectors at the national and county level. We collected quantitative data from secondary sources, including budgets and budget reports. We analysed qualitative data using the thematic approach and carried out descriptive analyses on quantitative data. The budget execution processes within counties in Kenya were characterized by poor budget credibility, cash disbursement delays, limited provider autonomy and poor procurement practices. These challenges were linked to an inappropriate input mix that compromised the capacity of county health systems to deliver health-care services, misalignment between county health needs and the use of resources, reduced staff motivation and productivity, procurement inefficiencies and reduced county accountability for finances and performance. The efficiency of county health systems in Kenya can be enhanced by improving budget credibility, cash disbursement processes, procurement processes and provider autonomy.

**Key messages**

- Poor budget credibility compounded by cash disbursement delays limits service delivery thereby limiting health system efficiency.
- Limited involvement of the health department during execution of the budget reduced staff motivation and limited accountability.
- Poor procurement practices led to reduced value for health system resources.
- Rigid controls limited budget execution and provider autonomy.

## Introduction

Achieving universal health coverage goals depends on allocating sufficient resources to health and using the allocated resources efficiently (Barroy *et al.*, 2016). There are two types of efficiency: (1) allocative efficiency which entails having the best input combination for output maximization (Schick, 1998) and (2) technical efficiency which means getting maximum outputs for available inputs or using the least possible inputs for a given set of outcomes (Schick, 1998). Inefficiencies within the health sector result in the wastage of 20–40% of health resources (World Health Organization, 2010). Improving efficiency is an important source of increased health resources (Barroy *et al.*, 2016; 2018).

Several studies have identified public financial management (PFM) processes as a driver of health system efficiency (Piatti-Fünfkirchen and Schneider, 2018; Zeng *et al.*, 2022). PFM refers to mechanisms that governments have in place to collect, allocate, use and account for public resources. PFM happens within the budget cycle—budget formulation and approval, execution and evaluation. Our previous paper discusses how the budget formulation structures and processes influence the efficiency of county health systems (Musiega *et al.*, 2022). This paper focusses on the relationship between budget execution and the efficiency of county health systems.

Budget execution encompasses the provision of promised revenues and the use of these resources to achieve health system objectives (Piatti-Fünfkirchen and Schneider, 2018). While there are challenges across all three aspects of the budget process in low- and middle-income countries (LMICs), the problems are worse downstream (at budget execution and evaluation) (World Health Organization [WHO], 2016; Barroy *et al.*, 2019). Government budgets are better made than they are executed (World Health Organization, 2018). While it is difficult to execute a poorly formulated budget, it is possible to poorly execute a well-formulated budget (Piatti-Fünfkirchen *et al.*, 2021). Well-formulated budgets that incorporate society’s views and health system needs are of no value if they are not implemented (Piatti-Fünfkirchen *et al.*, 2021). The budget execution processes must ensure that budgets are well implemented. Several countries have reported challenges with their budget execution processes including poor budget credibility—governments fail to honour budgetary commitments; poor budget absorption—departments of health fail to utilize funds availed to them; corruption—embezzlement of public funds and misappropriation of funds—use of public funds for purposes other than those that were intended (Asante *et al.*, 2006; Piatti-Fünfkirchen and Schneider, 2018). All these challenges can likely compromise the efficiency of the health sector (Piatti-Fünfkirchen *et al.*, 2021).

**Figure 1.**
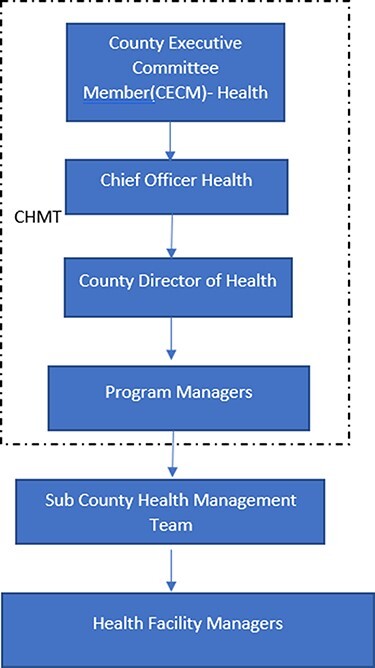
County Department of Health Organization Structure

Kenya has a devolved government with a national government and 47 county governments (Constitution of Kenya, 2010). County governments are responsible for health service delivery and other devolved functions. The county governments are funded by exchequer allocations, own source revenue collections, grants and donors with exchequer allocations forming the highest percentage of county revenues. Counties are responsible for allocating, planning, implementing and accounting for budgets at their level (Tsofa *et al.*, 2017). Each county has an executive and a legislature (county assembly) to enable them to fulfil their mandate. The county assembly is made up of elected and nominated ward representatives and has a legislative and oversight role. The county executive committee (CEC) is chaired by the governor and has appointed members who double up as heads of county departments. The CEC allocates and manages the county resources. All departments also have chief officers who are the accounting officers for their departments and report to their respective CEC members. The chief officer of finance is the overall county accounting officer. The chief officer of finance manages and supports departmental chief officers including the chief officer of health to execute their budgets. The health budget execution is executed by various entities including the department of finance, the county health management team (CHMT), the sub-CHMTs and health facilities (Figure 1).

Kenya, like other LMICs, Kenya has reported challenges in the budget execution process that can likely compromise efficiency. For example, at the national level, one study reported poor budget absorption, poor procurement practices and cash disbursement delays (Glenngård and Maina, 2007). At the county level, studies have reported reduced provider autonomy (E. W. Barasa *et al.*, 2017), inadequate budget allocation (Mbau *et al.*, 2018) and *ad hoc* reallocation of health resources during execution (Waithaka *et al.*, 2018). Understanding how the budget execution structures and processes interact to influence the efficiency of the health system is an important research question.

This study is part of a larger, phased study that examined the efficiency of county health systems in Kenya (the Kenya Efficiency Study). Phase one of the study examined and reported stakeholder perceptions about the factors that affect the efficiency of county health systems in Kenya and identified PFM as one of those factors (Nyawira *et al.*, 2021). Phase two measured the level and determinants of county health system efficiency. This phase ranked the 47 counties using an efficiency score and identified the absorption of county budgets (a budget execution issue) as a determinant of county health system technical efficiency (Barasa *et al.*, 2021). This paper reports part of the findings of the third and final phase of the study, which entailed in-depth case studies in selected counties to examine identified determinants of county health system efficiency. Specifically, in this study, we examine how the budget execution process influences the efficiency of county health systems in Kenya.

## Methods

### Conceptual framework

**Figure 2.**
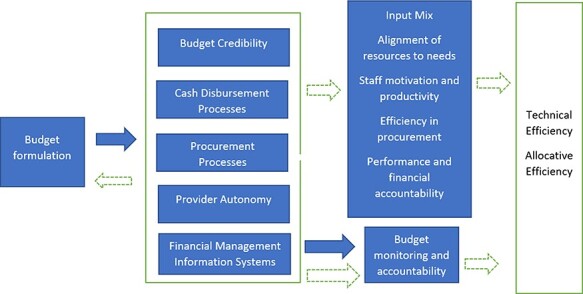
Conceptual framework

We developed a conceptual framework (Figure 2) from a scoping review on the effects of budget execution processes on the efficiency of health systems. We found five potential dimensions of the budget execution process that may influence the efficiency of health systems: budget credibility, cash disbursement processes, procurement processes, provider autonomy and financial management information systems. These dimensions influenced the efficiency of health systems by influencing the input mix, alignment between county needs and use of resources, staff motivation and productivity, efficiency in procurement practices and accountability. This conceptual framework guided the development of tools and data analysis.

### Study design

We conducted a mixed methods case study with data collected through in-depth interviews and document reviews. The case studies allowed for the exploration of the budget execution process within counties in Kenya. We used qualitative methods to explore stakeholder perceptions and quantitative methods to analyse budget data to contextualize the qualitative data.

### Study cases

We selected four cases—counties—using the level of technical efficiency reported in phase two of the Kenya Efficiency Study (Barasa *et al.*, 2021). This study employed data envelopment analysis with county health expenditure, number of health workers and number of health facilities as inputs and disability adjusted life years as the outcome of county health system (Barasa *et al.*, 2021). We classified counties with efficiency score above 0.9 as efficient and those below 0.5 as inefficient. From the group of inefficient and efficient, we purposefully selected two counties each to participate in the study taking into consideration other determinants of efficiency such as geographical distribution and prevalence of human immunodeficiency virus (HIV)/Acquired immunodeficiency syndrome. That is, we selected counties with varying population size and HIV prevalence. Counties A and B were the selected efficient counties, and Counties C and D were the inefficient counties (Table 1).

**Table 1.** County profiles

County	Efficiency score^a^	Population (2019)	Total county public health recurrent expenditure (2018/2019) KES	Total county public health development expenditure (2018/2019) KES	Per capita county public health expenditure (2018/2019) KES	Percentage of public facilities	Percentage of private facilities
A	0.9	1 163 186	1,884,620,000.00	46 490 000.00	1660.190202	62%	38%
B	0.9	990 341	949 629 480	615 371 170	1580.264424	47%	53%
C	0.4	1 131 950	2 121 046 189	370 754 248	2201.334367	63%	37%
D	0.5	315 943	749 054 078	167 564 044	2901.21358	71%	29%

a the efficiency score was computed using data envelopment analysis. The measures represent relative efficiency of county health system and have a range of 0–1.

### Study population

At the county level, we collected data from the county department of health and the county treasury and finance. Within the department of health, we collected data from the county level, the sub-county level and health facilities including hospitals and primary health-care centres. Within the department of finance, we collected data from the administration and the finance and procurement offices. At the national level, we collected data from the national treasury and the ministry of health and development partners. We collected data using in-depth interviews and document reviews. Some entities such as the auditor general and controller of budget did not respond to our request for an interview, but we included publicly available reports from them in the study.

### In-depth interviews

We conducted the in-depth interviews using topic guides developed from the study’s conceptual framework. We piloted the interview tools with county-level respondents from a county that was not part of the main study. We purposively selected 70 participants (Table 2) directly involved in county health budget planning, execution and accountability. The participants were selected to ensure a balance between counties and to allow the inclusion of the various levels at which the health budget is planned, executed and monitored. All county-level interviews were conducted at a private place at the respondent’s workstation or an alternative place based on the respondent’s preference. Some national-level interviews were conducted online because of respondents’ preferences. Interviews were audio-recorded and took between 45 and 90 min. Study respondents provided signed informed consent.

**Table 2.** Study participants profile

	County-level respondents				
Interviewee group	A	B	C	D	National-level respondents
Health sector	4	6	3	3	1
Finance sector	4	1	2	5	1
Sub county health managers	0	3	2	0	–
Health facility managers	7	9	5	9	–
Donors	–	–	–	–	6
Subtotals	15	19	12	17	8
Total	70				

### Document reviews

We collated and analysed documents related to budget execution (Table 3). These documents included national-level policies and guidelines that relate to budget execution. At the county level, we collected budget execution documents from each study county. County A did not provide its votebooks, and hence we used data reported to the controller of budget in place of data from votebooks.

**Table 3.** Documents reviewed

Documents type	Total reviewed
County Votebooks 17/18	3 (one from each county)
County Votebooks 18/19	3 (one from each county)
County Fiscal Strategy Paper 18/19	4 (one from each county)
County Budget Review and Outlook Paper 18/19	1 (contains consolidated data from all counties)
County Budget Operationalization Manual	1 (national guideline used by all counties)
Public Finance Management Act	1 (national guideline used by all counties)
Public Finance Management Guidelines	1 (national guideline used by all counties)
County Governments Budget Implementation Review Reports	1 (contains consolidated data from all counties)
County Audit Reports FY 18/19	4 (one from each county)
Total	19

### Data analysis

We transcribed all the recordings and transferred the data to NVIVO for analysis. We analysed the qualitative data using a thematic approach that entailed developing a pre-analysis theme followed by identification, organization, description, analysis and reporting of themes found in a data set (Braun and Clarke, 2006). We conducted descriptive analysis of budget data on Microsoft excel. We used quantitative data to contextualize the results of the qualitative data.

## Results

In this section, we first present the budget execution process within county health systems in Kenya; then, we present the following five dimensions of the study’s conceptual framework (Figure 1): (1) budget credibility, (2) cash disbursement process, (3) procurement process, (4) provider autonomy and (5) financial management information system. Summary findings per county are outlined in Table 4.

**Table 4.** Summary findings per county

Issue	County A	County B	County C	County D
Honouring of budget releases (from national treasury)	Fully honoured	Fully honoured	Fully honoured	Fully honoured
Budget implemented as per plans developed	Executed as per plans	Not executed as planned	Not executed as planned	Not executed as planned
Timely payment for goods and services	Timely	Delayed	Delayed	Delayed
Competitive bidding for tenders	Competitive	Not competitive	Not competitive	Not competitive
Procurement process delivers goods and services timely	Timely	Delayed	Delayed	Delayed
CDOH gets value for money from procured goods and services	Value for money	No value for money	No value for money	No value for money
Frontline workers involved in procurement	Partially involved	Not involved	Partially involved	Not involved
Provider financial autonomy over own source revenue	Partial autonomy	Partial autonomy (until October 2020)	No autonomy	No autonomy
Access to IFMIS granted to the CDOH	Departmental access	Departmental access	Departmental access	No departmental access

### Budget execution process

**Figure 3.**
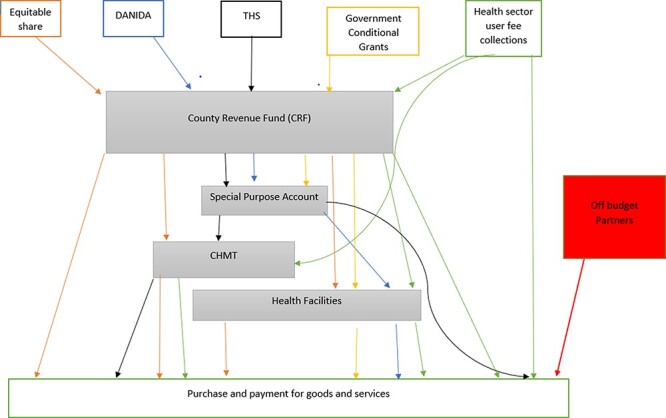
Budget execution points and funds flow processes within county health systems

There was no standard approach to executing health budgets within county health systems in Kenya. The county government received revenue from multiple sources and used the revenue through multiple channels. There were five revenue sources; equitable share allocation by the national government, on-budget donor conditional grants from the Danish International Development Agency (DANIDA) and the World Bank’s Transforming Health Systems project (THS), government conditional grants (user fee forgone and tertiary hospitals [Level 5] grants), own source revenue (user fees collected and insurance reimbursements) and off-budget partner support. Expenditure of the collected revenue also took place at multiple levels including county treasury (the county revenue fund [CRF] and special purpose account), CHMT and health facilities and at partner level by off-budget partners (Figure 3).

### Budget credibility

Budget credibility refers to the extent to which governments honoured their budgetary promises. It influenced county health system efficiency through (1) timely realization of expected revenue for budget execution and (2) the implementation of the budget as per plans developed during formulation.

#### Despite counties receiving their full equitable share, they did not always disburse budgets allocated to county departments of health

County departments of health (CDOH) respondents linked the failure to honour approved budgets to (1) county failure to realize revenue targets from some sources (Table 5), (2) limited transparency in the budget execution process, (3) minimal departmental control over resources and (4) failure to meet conditions attached to conditional grants:

**Table 5.** Actual county budget receipts as a percentage of budget allocation

	Percentage released							
	County A		County B		County C		County D	
Source of fund	2017/2018	2018/2019	2017/2018	2018/2019	2017/2018	2018/2019	2017/2018	2018/2019
Equitable share	100%	99.83%	100%	100%	100%	100%	100%	100%
Own Source revenue	84.39%	76.57%	61.51%	149%	90.12%	58%	145.70%	106%
THS	31.20%	47.30%	31.30%	40.90%	111%	48.10%	45.50%	51.10%
DANIDA Grant	47.00%	143.10%	64.50%	100%	100%	100%	100%	100%
User fee forgone grant	106.40%	100%	94.90%	100%	51%	100%	50.20%	100%

Source*: County Governments Annual Budget Implementation Review Reports (2017/2018, 2018/2019).*

‘*we don’t get money as a chunk from the national treasury it trickles in periodically. It is then the department of finance that decides how much to give to health from that disbursement. So budgeting is one thing, but executing that budget in the context of the counties is very difficult’* County Health Manager County B

Failure to receive disbursements of expected revenue influenced efficiency in various ways. First, CDOHs had to forfeit key budget items. This compromised the health system input mix and compromised the capacity of facilities to provide health services.

*‘We included the repair of our faulty solar system in the budget. However, it was not funded. During blackouts, we are forced to conduct maternal deliveries in darkness. The mothers leave the hospital with a negative perception. They discourage other mothers from coming to the hospital. We put a lot of effort in mobilizing mothers to deliver at the hospital but they don’t come because we have challenges’* Facility Manager County D

Second, failure to realize expected revenue resulted in late payment and non-payment of bills from private suppliers of health commodities (Table 6). This led to private suppliers subsequently refusing to supply health commodities to the counties.

**Table 6.** Summary of total county pending bills (KES Millions)

	2017/2018			2018/2019		
	Development	Recurrent	Total	Development	Recurrent	Total
County A	84.15	235.2	319.35	51.73	229.8	281.53
County B	313.3	468.28	781.58	421.71	317.51	739.22
County C	666.21	80.27	746.48	949.74	89.7	1039.44
County D	853.44	95.59	949.03	891.6	490.51	1382.11
National Total	28 055.38	80 356.05	108 411.43	11 626.34	22 911.73	34 538.07
National Average	596.92	1709.70	2306.63	247.37	487.48	734.85

Source*: Controller of Budget.*

*‘Our Local Service Order (LSO) are never honoured because disbursements were not honoured. Yet the goods/services were delivered. This results in debts. Because of this, some of our suppliers decline to furnish our orders’*. Facility Health Manager County B

Third, it compromised the ability of the CDOH management to hold health facility managers accountable for performance. When disbursements to health facilities were not honoured, the CDOH supervisors lacked the legitimacy to supervise to ensure delivery of services.

*‘Supervision is very weak in health. The CHMT is embarrassed to visit health facilities to supervise what they’ve not funded. Besides if someone came here to supervise me, I would be very reluctant, and I believe any MOH (Medical Officer of Health) would be very reluctant to be supervised on what has not been funded despite budgeting and approval’* Facility Health Manager County C

### The budget was not implemented as per the plans developed during budget formulation

In three of the four study counties, utilization of funds deviated from the formulated budgets and plans. The CDOH respondents noted that the Controller of Budget approved expenditure based on requests that were directly linked to approved budgets. However, once these funds were availed, they were used at the discretion of the county treasury.

Respondents noted that budget execution that deviated from existing budgets and plans without a clear need for reallocation influenced efficiency in several ways. First, expenditure deviated from the county needs, compromising the achievement of health system targets.

*‘One can easily come up with a plan which is neither in the budget nor in the AWP. Our plans are made to achieve targeted indicators. So, if you conduct an activity that is outside your plan, you may not be working towards the intended goal’* County Health Manager County D

Second, unapproved deviation from the existing plans and budgets limited accountability. Departments spending the money were unable to track expenditures and verify service delivery.

### Cash disbursement processes

CDOH and treasury respondents in County A noted that their cash disbursement processes were well organized and timely, while respondents from the other three study counties noted that their processes were bureaucratic and late. The challenges with cash disbursement processes influenced efficiency by influencing (1) the timeliness of payment and (2) how payments were prioritized.

#### In three of the four counties (B, C and D), cash disbursements were late

Lateness in the disbursement of finances hindered efficiency in several ways. First, it compromised service delivery and hence potentially negatively affected health outcomes.

*‘Our performance depends on the flow of funds. As the CEC I have a performance contract that should be executed within specified timelines… But the flow of funds has been the greatest challenge. Because if funds don’t flow, you may plan but you can’t implement’* County Health Manager County B

Second, some disbursements came too late in the financial year influencing the ability to honour commitments for already incurred expenses (Table 7), absorption for un-incurred expenses (Table 4) and ultimately resulting in pending bills (Table 8).

**Table 7.** County department of health total outstanding commitments at the end of financial year 2018/2019

Details	County A	County B	County C	County D
Recurrent outstanding commitments	15 627 958.00	11 920 166.00	0	4 888 500.00
Development outstanding commitments	37 629 926.00	998 130.00	161 442 285.00	519 535.00
Total outstanding commitments	53 257 884.00	12 918 296.00	161 442 285.00	5 408 035.00
Outstanding commitments as a percentage of total commitments	2.7%	2.4%	6.5%	0.6%

Source*: Office of the controller of budget.*

**Table 8.** County health budget absorption financial year 2018/2019

Details	County A	County B	County C	County D
Budget absorption recurrent	97%	98%	101%	63%
Budget absorption development	34%	86%	77%	104%
Total budget absorption	89%	98%	97%	68%

Source*: Office of the controller of budget.*

*‘The unutilized funds will be the opening balance for the next financial year. So your budget for the next financial year will be financed less the unutilized balance available in the account’* Facility Health Manager County D

Third, in the three counties, it resulted in delays in the payment of staff salaries which resulted in staff demotivation that, among others, manifested in absenteeism. In County A, timely payment of salaries ensured staff reported to work on time.

*‘Salary delays have forced health workers to look for alternative sources of income. These are maintained as a cushion once salaries are paid. But they have a negative impact on the health system. The health workers’ priorities shift resulting in absenteeism thereby lowering performance’.* County Health Manager County B

*‘Yes, timely cash disbursement affects us positively the fact that it is not delaying, of course, there is no reason for staff to report on duty or maybe have so many issues’* County Health Manager County A

#### In all four counties, there were unclear mechanisms for priority setting during cash disbursement

It was at the discretion of treasury accountants to decide who to pay first. This had several implications for efficiency. First, the payments reflected neither the health managers’ nor the patients’ needs. At the health facility level, the health facility managers felt demotivated. They reported that they often worked hard to raise revenue just for the money to be used in unclear ways.

*‘At both the department and county level, accountants have the ultimate say. As a director, I do not know the requisitions my accounting officer has sent to treasury for payment. The accounting officer is not a medic. My priorities are not their priorities. So you end up with a very distorted payment schedule that does not address the patient needs’* County Health Manager County D

Second, it introduced corruption in the payment process as suppliers were forced to lobby for payment. This reduced competitive bidding as only suppliers who were able to lobby worked with the county.

*‘There are several complaints about payments of the suppliers within the CDOH. If the challenges are experienced frequently, then pending bills accumulate. This forces people to prioritize which in turn results in lobbying. Those who are able to lobby are given first preference but there are other small suppliers who don’t have the muscles to lobby’* County Finance Officer County B

### Procurement and supply chain

The respondents noted that the procurement process influenced the efficiency of health systems in various ways: (1) competitive awarding of tenders, (2) timeliness of the procurement process, (3) value for money, (4) frontline workers’ involvement in the procurement of goods and services and (5) use of Kenya Medical Supplies Agency (KEMSA) as a single supplier.

#### Respondents in three of the four counties noted that tenders were not competitively awarded

For example, in Counties B and D, contracts were awarded based on political patronage. In County C, contracts were awarded to companies that could pay kickbacks. This had several implications on efficiency. One, contracts were awarded to companies that were not qualified. This resulted in delivery of substandard goods and services including buildings which would be uninhabitable <5 years after their completion.

*‘In my opinion, county contracts are not awarded competitively rather, on political terms. As a result, most of the goods and services delivered are substandard. They build structures without toilets or running water. How can a theatre operate without running water?’* Facility Health Manager County D

Second, it was felt that failure to award tenders competitively exacerbated the problem of misplaced priorities during payment. Interview respondents reported that people who had links to government officials were more likely to be paid first. This resulted in a situation where neither the county nor the health workers were interested in patient needs and experiences.

*‘Some of the suppliers are relatives to the politicians so if you are a relative or friend you’ll be paid. But the others are not paid. Whether the suppliers supply or not, we don’t get involved. When they fail to supply food, patients either buy or their relatives bring food from home. Nobody cares’.* Sub County Health Manager County B

Third, it reduced accountability over services and goods procured. Because the suppliers had connections, it was impossible for the health workers to condemn substandard goods or to raise issues over the quality of the goods or services. The health workers faced sanctions when they raised questions about suppliers. Besides, suppliers were still paid even after supplying substandard goods and services.

*‘According to the public procurement act, procurement should be competitive. However, the top management interferes with the process. It might look competitive on paper because they don’t want to be in problems, but it is not. If it was competitive and the contractor offers substandard services, then the county will not pay, but if they are offering substandard services and they are still paid it means there is an influence’* Sub County Health Manager County B

#### Respondents in Counties B, C and D noted that procurement processes were bureaucratic, lengthy and characterized by delays

The lengthy bureaucratic process influenced efficiency in several ways. First, it resulted in delayed delivery of services. For example, in County C, it was reported that the lengthy procurement process delayed services even when the resources were available. In County A, considerations were put in place to ensure timely procurement for service delivery.

*‘Delivery takes too long. This has affected service delivery. Currently, at the county referral hospital patients are buying everything, including gloves, needles for anesthesia, betadine for cleaning the operation site before an emergency CS, sutures for stitching after surgery, everything. If the patients do not have money, then we don’t do the procedure’* Facility Health Manager County C

*‘Time is of the essence. When there are procurement delays they result in service delivery delays. So we try to improve time….in the long run we have patients waiting for the services or medicines, we normally make sure that when we’re purchasing drugs in KEMSA we ensure that time is taken care of so that we can deliver services to the public’.* County Finance Officer, County A

Second, because of the long procurement process, then suppliers overpriced goods and services because they anticipated that the procurement process will consume more time and resources.

*‘Goods and services are overpriced because of the expected delay in payment. I may order in July then they’re delivered in December. Suppliers adjust for inflation and the bureaucracies of getting a tender thereby increasing the cost’.* County Health Manager County C

Third, health workers in emergencies and small health facilities were forced to contravene the procurement laws; because the process would increase the cost and timeliness of acquisition, they opted for direct procurement. This direct procurement created loopholes for misappropriation of funds, especially when left unmonitored.

*‘We procure small quantities so we avoid the policy of procurement protocols. What we have is little, taking it through procurement would not make sense. We do not do tendering and vetting; we just buy locally’.* Facility Health Manager County C

#### The procurement process did not result in value for money

Value for money was compromised in two ways. First, in some instances, the counties paid for poor-quality or undelivered goods. Health workers were forced to work with poor-quality products which still incurred the market price for high-quality goods:

*‘We have specifications that have to be met. Previously, I almost lost my job because I rejected reagents worth millions of shillings. The reagents did not meet our specifications neither did the supplier maintain cold chain during transportation as required. I was condemned but I stood my ground. The supplier returned the goods. They were to replace but I don’t know whether or not it was replaced but the supply was paid’* County Health Manager County C

Second, the government paid more than the market price for commodities.

*‘It doesn’t give value for money because sometimes the quotation made for renovating a building is worth another building’.* Sub County Health Manager County C

*‘Whatever budget we have is less than what it should be. We can have a billion shillings but in terms of worth, it is five hundred million. You end up doing very few things at a very high price. The budget and outcomes do not correlate’* County Health Manager County B

#### In two of the four counties, frontline users of goods and services were either not involved or inadequately involved in procurement

While the procurement process requires that frontline staff are involved in key steps of the procurement process, this did not always happen. Failure to involve the users in the procurement process led to the procurement of items that did not meet the user’s expectations.

*‘Neither the CDOH nor the health facility managers are consulted prior to commencing construction. We come in during inspection. Health facility managers find buildings coming up, when they question, they are told to mind their business. The building will be completed then condemned at inspection’.* County Health Manager County B

#### Counties were required to procure from KEMSA as their first source for medicines and medical products

KEMSA is the country’s central public procurement and supplies agency for health-care commodities. The requirement for counties to exclusively procure from KEMSA had both positive and negative implications for efficiency. Unlike private suppliers, KEMSA sustained supplies during cash disbursement delays. This enabled continuity of service provision, thereby enhancing efficiency.

*‘we delayed paying KEMSA because of the delay in the disbursement of funds from the national treasury. The CEC persuaded KEMSA to supply us and they agreed despite us owing them 19 million’.* County Finance Manager County B

Second, KEMSA also offered cheaper prices than some of the local suppliers, thereby providing value for money.

*‘KEMSA provides value for money because they supply at the quoted price. The prices and quantities are clearly outlined. When you procure from other traders you may not realise value for money, their pricing is a bit crazy. The cost per unit pack is higher’.* Facility Health Manager County D

However, KEMSA compromised efficiency when they were unable to supply all the required medicines and supplies, leading to interruptions in health service delivery.

*‘KEMSA doesn’t stock lab reagents. They cannot fully furnish our requests. They only provide grouping reagents, stool containers, and urine containers. Yet we cannot work without reagents’.* County Health Manager County B

### Provider autonomy

#### Health facilities in all four counties had either partial autonomy or no autonomy over their own source revenue

PFM rules required that all revenues generated are sent to the CRF rather than retained in health facility accounts. While both the respondents and the document review indicated that there were existing legal provisions for health facilities to retain their revenue, most counties were unwilling to explore this.

*‘All revenue generated by the CDOH goes back to the county. For a long time finance was quoting the PFM act insisting that they require a law for CDOH to use its revenue at source. We finally obliged and created the law that was passed by the assembly. But, it has not been operationalized to date’* County Health Manager County B

*‘Patients are dissatisfied with our services because we do not have funds at the facility. Ideally the money should go to the CRF, but it should be sent back to the health facilities in less than 15 days but that has not happened since October’.* County Health Manager County B

Lack of financial autonomy, whether partial or complete, had several implications on efficiency. First, health facilities had constrained access to resources. As a result, the health system was unable to adequately deliver health services.

*‘The County government used to purchase supplies for lab and radiology. But they no longer purchase. We buy all these supplies. They consume a lot of our money. We charge patients for laboratory and radiology services. The county collects all the money. Then we are left without resources to replenish supplies’* Facility Health Manager County A

Second, the process for health facilities to access funds within the CRF was long and bureaucratic, resulting in delays in the provision of health services.

*‘In case of an emergency, you might lose a patient because you lack essential commodities. And this happens frequently. That is the downfall of a health system that is not properly financed’* Sub County Health Manager County B

Third, limited autonomy compromised the link between financial and performance accountability. Health facilities were unable to effectively evaluate their performance as they were unaware of the full extent of their expenditure.

*‘You see there is what the county spends on the facility and then there is what I spend. My expenditure comes from NHIF reimbursement. This I can evaluate what worked and what didn’t work, and improve on subsequent expenditures. But it’s hard to evaluate what the county probably spent on us’.* Facility Health Manager County A

#### In three of the four counties, the health department’s control over the procurement process was limited

Power over what should be procured, and when, was with the county treasury. This compromised efficiency by limiting service delivery and misaligning procured commodities with needs at the health facility level.

*‘Finance issued a tender to purchase an anesthetic machine. The supplier was unable to get the specifications requested in the tender. He came to consult us, only to realize, the user, the anesthetist wasn’t consulted on the specifications. Going forward, we want the users to decide what is to be bought. This decision should not be left to anyone who has access to the money’* County Health Manager County B

### Financial management system

County governments used the integrated financial management system (IFMIS) to process all government payments. The use of this system was rolled out in 2018 to enhance the accountability of funds. This system has had several effects on the efficiency of the county health system. Respondents noted that IFMIS has traceability and hence improved financial accountability. Respondents also felt that IFMIS also improved efficiency by digitizing PFM processes. However, this was limited by the requirement that counties provide printed documents to the national government for approval.

#### Access to the IFMIS was limited to a few people, and even amongst the few people, there were varying levels of access

As a result, it has limited access to information thereby exposing health funds to misuse and limiting the departments’ autonomy over their resources.

*‘the chief officer finance has more powers in the system, he can reallocate budgets between departments. There are instances when we request to execute part of our budget, but the request is declined because the allocation is exhausted. Yet the CDOH did not spend the resources. My chief officer cannot claim with certainty that we have resources on a certain vote. IFMIS payment powers should be devolved to the departmental level’.* County Health Manager County B

## Discussion

We examined the relationship between the budget execution process and the efficiency of county health systems in Kenya. We found that each dimension of the budget execution process has potential effects on health system efficiency. For the county health departments in Kenya, the budget execution process defined the implementation of their work plans and almost all service delivery activities.

We set out to explore the relationship between budget execution processes and efficiency in two efficient and two inefficient counties. Challenges within the PFM system were generally cross-cutting, with no clear distinction between efficient and inefficient counties. However, one county stood out. County A, one of the efficient counties, had more credible budgets, more efficient cash disbursement processes, partial provider autonomy and more efficient procurement systems. This perhaps provides some evidence that effective PFM processes enhance the efficiency of health systems. However, while County B was ranked as efficient, we found that it shared similar PFM challenges with the inefficient counties. This mixed finding could be because the nature of PFM practices documented is perverse in Kenyan counties, with differences in degrees across countries that are difficult to tease out using a qualitative approach. It could also be because the counties that were ranked as efficient by the quantitative analysis by being on the efficiency frontier are inefficient in absolute terms, even though they are relatively more efficient than the counties that are at a distance from the efficiency frontier. This notwithstanding, the study found that county budget execution challenges could potentially influence the efficiency of the county health system in several ways.

First, some budget execution practices are likely to compromise the input mix of county health systems with negative impacts on the capacity of county health systems to deliver health-care services and consequently health outcomes. We found that county health budgets were hardly credible, characterized by the failure of county governments to honour budget allocations to county health departments and delays in the disbursement of funds. These delays and non-disbursement of funds thus constrained the resources available to county health department and reduced the county health department budget absorptive capacity. Delays in procurement also impacted negatively on service delivery. Studies from other settings have documented how the lack of credibility of budgets limits efficiency by disrupting service delivery and impairing manager’s ability to implement health system plans (Piatti-Fünfkirchen *et al.*, 2021). It has also been shown by other studies that cash disbursement delays may lead to rushed spending and poor budget absorption when funds are availed at the end of the financial year (Glenngård and Maina, 2007; Abekah-Nkrumah *et al.*, 2009; Piatti-Fünfkirchen *et al.*, 2021).

Delays in funds disbursement and procurement were also because of the limited financial and managerial autonomy of county health departments and health facilities. Other studies in Kenya have demonstrated that limited provider autonomy over managerial and financial roles hinder service delivery (Barasa *et al.*, 2017; de Geyndt, 2017). The main reason for the reduced autonomy of county health departments and health facilities is the requirement by the PFM laws for all funds to be managed centrally from the CRF account (Government of the Republic of Kenya, 2012). It has however been observed that it is possible to provide autonomy to county health departments and health facilities under existing PFM laws, suggesting that the lack of autonomy is an intentional misinterpretation of PFM laws informed by county leaders interest to control resources centrally. This study found that autonomy was additionally compromised by several practices, including limited access to the financial management information systems. Similar findings were reported in Tanzania where investment in a financial management information system that could be implemented to the lowest planning unit enhanced reporting and accountability, thereby enhancing health system efficiency (Piatti-Fünfkirchen and Schneider, 2018).

Second, inefficiencies could also arise due to misalignment between county health needs and the use of resources. We found that actual county department of health expenditures deviated from approved budgets. Further, we found that cash disbursements by county treasury accountants used unclear considerations, resulting in the prioritization of expenditures on activities that were not aligned with county health department priorities. In Kenya, one study reported the reallocation of health system resources to fund the governor’s promises (Waithaka *et al.*, 2018). In Democratic Republic of Congo, health funds were used to finance administrative activities in the office of the governor (le Gargasson *et al.*, 2014). Rent seeking and political patronage of procurement processes also contributed to the misalignment of payments and county department of health priorities. Misalignment also resulted from the inadequate involvement of frontline health workers in the procurement process for goods and services and the reduced financial and managerial autonomy of county health departments and health facilities.

Third, county health system efficiency could be negatively impacted by reduced staff motivation and productivity. We found that delays in funds disbursements resulted in late payment of staff salaries, leading to demotivation. Health facilities managers were also demotivated by the lack of alignment between their stated priorities as articulated in approved budgets and plans, and the county treasury accountants revealed priorities by disbursing funds for specific activities. In Yemen and Ghana, cash disbursement delays led to halting of key activities and delays in salaries that demotivated employees (Asante *et al.*, 2006; Elgazzar, 2011).

Fourth, procurement inefficiencies, which included the procurement of substandard goods and services, and the inflation of procurement prices by supplies of goods and services, could negatively impact county health system efficiency. We found that county contracts were sometimes not competitively awarded and instead influenced by political patronage and bribes, sometimes resulting in substandard goods and services. Furthermore, suppliers of goods and services to counties often inflated procurement prices because of anticipated delays in payments by county governments. Similar findings are reported Czech Republic and South Africa where poor procurement practices resulted in inefficiencies (Global Access Partners, 2016; Munzhedzi, 2016; Transparency International Česká republika, 2016).

Finally, inefficiencies could arise from compromised county accountability for finances and performance. Staff were not empowered to reject substandard goods and services because of the political patronage enjoyed by suppliers. Accountability for performance was also compromised by the reduced financial and managerial autonomy of county departments of health and managers. Further, delays or non-disbursement of funds and the misalignment of county department of health’s plans and actual expenditures meant that county managers had reduced legitimacy to hold staff accountable for performance. In Tanzania and Zambia, reduced managerial autonomy limited their accountability over performance (Piatti-Fünfkirchen and Schneider, 2018a).

This study had several limitations. First, we only sampled 4 out of the 47 counties in Kenya. Budget execution processes within counties are diverse. Besides, given the semi-autonomous nature of counties in Kenya, their sources of revenue and laws are equally diverse. Second, we collected budget data from multiple documents at both county and national levels. Besides, the data were extracted at different time points. Where the data conflicted, we used the most complete data source.

Despite the limitations, county governments need to improve their budget execution processes to improve the health system efficiency. First, counties should make realistic own source revenue projections. This will ensure that the budget ceilings are realistic and set the ground for a credible budget. Second, counties should strive to meet the conditions attached to conditional grants to enhance the credibility of conditional grants. Third, the CDOH should be involved in prioritizing payments to ensure that payments reflect the needs of the departments. Fourth, the government should improve timeliness in the procurement process including timely payments; this will reduce the cost of goods and services and encourage competitiveness as more suppliers will be motivated to participate in the procurement process. Fifth, county governments should implement mechanisms to ensure that providers have more managerial and financial autonomy. Sixth, the government should increase provider autonomy over both their financial and managerial functions. This will possibly increase transparency in the budget execution process, align budget execution to health systems needs and increase health facility managers’ accountability for performance. Finally, the government should roll out IFMIS to the lowest planning unit. This will increase transparency and enhance accountability over resources, thereby enhancing efficiency.

### Conclusion

In conclusion, all the five aspects of budget execution influence health system efficiency directly or by influencing the budget formulation evaluation processes. While a well-formulated budget is a good starting point for efficiency within the health system, the budget should be well implemented to realize the desired outputs and outcomes. More research is required on various issues around budget execution for enhanced efficiency, including public participation during budget execution, the flexibility of health budgets in responding to emergencies, how procurement practices can be improved for enhanced value for money and the role of actors in budget execution.

## Data Availability

All the data are provided in the article as quotes and tables.

## Reflexivity statement

The study was conducted by researchers from both high income (K.H. and C.N.) and LMICs (A.M., B.T., L.N., R.N., J.M., A.M., S.M., I.M., J.J. and E.B.). The study team had seven women with three senior scientists (K.H., S.M. and J.J.); three upcoming scientists (A.M., L.N. and R.N.) and two policymakers (A.M. and I.M).. The study team comprised five men; three senior scientists (B.T., C.N. and E.B.); one upcoming scientist (J.M.) and one policy maker (A.M.).
