# Correction to: Examining the influence of budget execution processes on the efficiency of county health systems in Kenya

**DOI:** 10.1093/heapol/czad001

**Published:** 2023-01-11

This is a correction to: Anita Musiega, Benjamin Tsofa, Lizah Nyawira, Rebecca G Njuguna, Joshua Munywoki, Kara Hanson, Andrew Mulwa, Sassy Molyneux, Isabel Maina, Charles Normand, Julie Jemutai, Edwine Barasa, Examining the influence of budget execution processes on the efficiency of county health systems in Kenya, *Health Policy and Planning*, 2022; czac098, https://doi.org/10.1093/heapol/czac098

In the originally published version of this manuscript, authors’ affiliations were incorrect. The correct affiliations are as follows:

Anita Musiega^1,2,*^, Benjamin Tsofa^3^, Lizah Nyawira^1^, Rebecca G Njuguna^1^, Joshua Munywoki^1^, Kara Hanson^4^, Andrew Mulwa^5^, Sassy Molyneux^3^, Isabel Maina^6^, Charles Normand^7^, Julie Jemutai^1^ and Edwine Barasa^1,2,8^

1 Health Economics Research Unit, KEMRI-Wellcome Trust Research Programme, 197 Lenana Place, Lenana Road, P.O Box 43 460–00100, Nairobi, Kenya

2 Institute of Healthcare Management, Strathmore Business School, Strathmore University, Ole Sangale Road, P.O. Box 59 857–00200, Madaraka, Nairobi, Kenya

3 Health Systems and Research Ethics Department, KEMRI-Wellcome Trust Research Programme, P.O Box 230–8010, Kilifi, Kenya

4 Faculty of Public Health and Policy, London School of Hygiene and Tropical Medicine, Keppel Street, London WC1E 7HT, UK

5 Directorate of Medical Services, Preventive and Promotive Health, Ministry of Health, Afya House, Cathedral Road, P.O. Box 30 016–00100, Nairobi, Kenya

6 Health Financing Department, Ministry of Health, Afya House, Cathedral Road, P.O. Box 30 016–00100, Nairobi, Kenya

7 Centre for Health Policy and Management, Trinity College, The University of Dublin, Dublin, Ireland

8 Centre for Tropical Medicine and Global Health, Nuffield Department of Medicine, University of Oxford, The Peter Medawar Building for Pathogen Research, South Parks Road, Oxon, Oxford OX1 3SY, UK

These errors have now been corrected.

